# Determinants of quality of life improvements in anxiety and depressive disorders—A longitudinal study of inpatient psychotherapy

**DOI:** 10.3389/fpsyt.2022.937194

**Published:** 2022-12-15

**Authors:** Marion Freidl, Melanie Wegerer, Zsuzsa Litvan, Daniel König, Rainer W. Alexandrowicz, Filipe Portela-Millinger, Maria Gruber

**Affiliations:** ^1^Clinical Division of Social Psychiatry, Department of Psychiatry and Psychotherapy, Medical University of Vienna, Vienna, Austria; ^2^Institute of Psychology, Alpen-Adria-University Klagenfurt, Klagenfurt, Austria

**Keywords:** quality of life, inpatient psychotherapy, cognitive behavior therapy, anxiety disorder, depressive disorder, predictors, treatment outcome

## Abstract

**Background:**

Quality of life (QoL) is substantially impaired in patients with anxiety disorders (AD) and depressive disorders (DD) and improvements in symptom burden after psychotherapy are not always paralleled by similar improvements in QoL. So far, little is known about treatment outcome in terms of QoL and predictors of QoL improvements following inpatient psychotherapy with a focus on cognitive behavior therapy (CBT). The current study aimed at investigating the relationship between changes in symptoms and QoL across different life domains. Additionally, predictors of a positive treatment outcome were evaluated.

**Methods:**

122 patients with AD and/or DD undergoing an 8-weeks inpatient CBT program completed self-report measures of psychopathological symptoms and QoL at pre- and post-treatment. Mixed effects models were used to investigate changes, a confirmatory factor analysis was applied to analyze the latent factor structure of the anxiety sensitivity index and binary logistic regression analyses were performed for predictors of QoL improvements.

**Results:**

Patients showed moderate to strong decreases in anxious and depressive symptoms and moderate to strong improvements in general QoL, particularly in the psychological and physical QoL subdomains. Changes in symptom burden correlated most strongly with psychological and physical QoL. In addition, poor QoL before treatment and low levels of specific anxiety sensitivity symptoms (items 1 and 5) significantly predicted improvement in QoL.

**Conclusion:**

Patients with poor QoL who are not as inhibited to openly express their anxious feelings particularly benefit from inpatient psychotherapy (individual and group) to improve their QoL. In contrast, our research suggests that patients who are too anxious to openly express their nervousness should receive additional social skills training, more focused treatment to build sufficient self-confidence to better engage in the treatment program.

## Introduction

Anxiety disorder (AD) and depressive disorder (DD) are the most common psychiatric disorders, with AD affecting about 3.6% and DD about 4.4% of the world’s population (1). AD and DD are leading causes of disability worldwide and contribute substantially to the global burden of disease (1, 2). Both disorders are more prevalent in women (3), usually begin early in adolescence and young adulthood, and often take a recurrent or chronic course. AD and DD cause suffering in those affected and are associated with significant impairment in social and occupational functioning together with a reduced quality of life (QoL) (1, 4–7).

AD is characterized by uncontrollable fears or anxieties that cause distress, and depending on what the anxiety is directed at, different types of AD (e.g., generalized anxiety disorder, panic disorder, social anxiety disorder, and various phobia-related disorders). Fears can be useful in response to a threat but can be disabling in response to a neutral stimulus that is then perceived as threatening (fear learning) (8) and even when anxiety-related sensations or behaviors are feared and mistaken as dangerous (anxiety sensitivity) (9). Fear learning, anxiety sensitivity and the lack of fear extinction and therefore impaired behavioral control, are critical in the development of AD and influence cognitive functioning at multiple levels (10).

In DD, emotional well-being is impaired, which is associated with low self-esteem, decreased energy levels, and loss of interest or pleasure in normally enjoyable activities. Depression can be mild, moderate, or severe and can lead to at least suicide (1, 4). Depressive disorders also impair cognitive functioning (attention, memory, information processing, and decision making), resulting in decreased cognitive flexibility (ability to adapt goals and strategies to changing situations) and decreased executive function (ability to perform all actions required for a task) (11, 12).

In Beck’s cognitive model (5) anxiety and depression involve dysfunctional negative views of oneself, one’s world in general, and one’s future. Three levels of cognition are identified to be responsible for the persistence of anxiety and depression: (1) schemas (enduring structural representations of human experience that direct the evaluation of new experiences), (2) biased information processing (in depression, recall of negative self-related information or in anxiety, selective processing of threat, danger, and helplessness), (3) negative “automatic” thoughts, images and memories that perpetuate an adverse emotional state. At a neurophysiological level, individuals with AD and/or DD show increased activation of the subcortical amygdalohippocampal region in which negative emotions are generated and decreased activation of the higher-level frontal regions in which negative emotions are cognitively controlled (5, 11).

Cognitive behavior therapy (CBT) is an effective treatment for AD and DD (13). It is based on the theory that negative thinking, emotions and behavior are connected and can be changed. CBT helps to identify and challenge unhelpful thoughts and learn practical strategies to help, which might lead directly to positive changes in QoL. Through the psychotherapeutic process, it mediates how thinking affects mood and behavior and consquently to think less negatively about oneself. Empirical studies show the efficacy of CBT at the neurophysiological level with decreased activation of subcortical regions of the amygdalohippocampus and increased activation of frontal regions (5).

Positive changes in the cognitively mediated perception of QoL, despite possibly remaining clinical symptoms, are reflected in the subjectively perceived QoL. The subjective level of QoL is therefore an important indicator of response to psychotherapy. The effect of psychotherapy on reduction of symptoms in AD and DD is well established in literature (13–16). Previous psychotherapy outcome studies have predominantly focused on symptom reduction as the main outcome variable to measure treatment effects. However, increasing emphasis has been put on broadening the evaluation of treatment outcomes and complementing traditional symptom measurements with QoL assessments (17–20). Mental disorders generally have a detrimental effect on QoL, and patients with ADs (13) or DDs (19, 21) report strong reductions in QoL in several domains, especially when they have been diagnosed with comorbid AD and DD (22).

Even though previous studies have clearly shown a beneficial effect of psychotherapy on symptom reduction and improvements in QoL, evidence concerning the relationship between these two treatment goals is sparse and still rather inconclusive. In general, some studies have found that diagnostic measures for ADs and DDs do explain only a small proportion of the variance in QoL (23), thus demonstrating that patients’ QoL is also substantially influenced by factors other than symptom burden.

Results of prior studies on the benefits of psychotherapy for AD and DD, results have been mixed (24, 25). One study comparing patients with DD or AD undergoing group psychotherapy with or without booster sessions (26), found that symptoms improved in both groups, whereas a significant increase in the social relations domain was only reported for patients in the booster session group. Importantly, these results do also suggest that QoL should not be studied as an overall construct, but rather be divided into specific sub domains of QoL (26, 27).

In some AD or DD patients, psychotherapy sometimes fails to improve clinical symptoms and QoL (28), so that recent studies have tried to identify outcome predictors (29). Altogether, results of these studies have been inconclusive and predictors of psychotherapy are not fully understood. One study, for example, did not find any influence of patient characteristics such as age, sex, income, duration of illness, or baseline medication on whether patients with AD did or did not respond to CBT. Comorbid mental disorders, e.g., DD, did not predict a worse outcome (29). One further study reported that sex, age, and partnership were no significant predictors for treatment response. Rather, it found therapy motivation, level of education and depression at baseline to have significant impact on a positive treatment outcome after inpatient psychodynamic therapy (30). Lower illness severity was found to predict improvements of QoL after group CBT in another investigation but only small and short-term effects of CBT on QoL, especially for social functioning, were found (31). Finally, improvements in QoL were not only seen as consequence of symptom decrease after psychotherapy, but also to as predict a subsequent improvement in depressive symptoms. Evidently a complex bidirectional interaction between symptom burden and QoL exists (18).

The role of anxiety sensitivity for QoL in AD and DD has not yet been sufficiently studied. Only few studies have examined the influence of anxiety sensitivity on QoL improvement after CBT. A possible influence of anxiety sensitivity on QoL improvements in AD (9, 32, 33) was shown and one study demonstrated a role of AS in predicting treatment response to a CBT program for perfectionism in patients with AD/DD and eating disorders (34).

In summary, more research is clearly needed to better understand the relationships between changes in psychopathological (i.e., anxious and depressive) symptoms and changes in QoL and to find potential predictors of QoL improvements after psychotherapy in AD and DD and the current longitudinal study contributes to this effort. To our knowledge this is the first study to examine a possible influence of anxiety sensitivity on treatment response as measured by QoL in a sample of AD and/or DD patients.

This study included patients with ADs and/or DDs who attended an 8-weeks inpatient CBT program and measured psychopathological symptoms and QoL at pre- and post-treatment. The aim was to evaluate changes in QoL and/or psychopathological symptoms and to find predictors for changes of QoL. We expected to find reductions in psychopathological symptoms and improvements of QoL and to find associations between these domains. One assumption was that these associations would be particularly strong for the psychological and less for the physical, social and environmental domains. Sociodemographic factors, psychiatric comorbidity, somatic complaints, symptom change, general QoL prior to therapy and anxiety sensitivity were investigated as possible predictors.

## Materials and methods

### Participants

152 patients aged 18–71 years who were seeking treatment at the inpatient behavioral therapy unit at the Medical University of Vienna from November 2013 to July 2016 were enrolled. Prior to admission, all patients underwent a pre-screening procedure to check therapy motivation and inclusion criteria. Therefore, the patients were clinically assessed by psychiatric residents or psychiatric consultants. Inclusion criteria for this study were an age >18 years, the diagnosis of an anxiety disorder (AD), depressive disorder (DD) or for both (AD/DD) according to the International Statistical Classification of Diseases and Related Health Problems (ICD-10) (35) and sufficient German language skills. Exclusion criteria were bipolar disorders, psychotic disorders, severe depressive disorders with psychotic symptoms or acute suicidality, severe cognitive impairment with serious impairment or inability to communicate, intellectual disability, severe substance dependency or/and acute intoxication and acute somatic illness.

### Procedure and treatment

The intensive 8-weeks therapy included various treatment elements: 12 sessions of group CBT (with a maximum of 8 patients) with a focus on AD or DD (36, 37). Group CBT included psychoeducation, teaching of coping skills, cognitive restructuring, exposure exercise, engaging in positive activities, as well as teaching of interpersonal competences as core elements and were based on the CBT manuals of (36–40).

These manuals give, among other things instructions for improved social skills and crisis management (37) as well as for the development of good therapeutic relationships.

Patients attended two individual CBT-sessions per week and accompanying occupational therapy, physical training and pharmacotherapy according to current treatment guidelines. Additionally, mindfulness training was offered once a week. At the beginning and the end of treatment, patients underwent an extensive psychiatric examination and were diagnosed according to ICD-10 (35) and completed self-assessment questionnaires asking on psychopathological symptoms, quality of life (QoL) and anxiety sensitivity (instruments described below). Data obtained were analyzed for the current study. The study was approved by the ethics committee of the Medical University of Vienna.

### Instruments

*Depressive symptoms* were assessed using the German version of the Beck Depression Inventory (21 items, scale scores 0–63) [BDI (41)]. A total BDI score of 0–9 is considered not depressed, 10–18 indicates mild-moderate depression, 19–29 indicates moderate-severe depression and 30–63 indicates severe depression. *Anxiety symptoms* were measured using the trait scale of the German version of the State-Trait Anxiety Inventory (STAI-T, 20 items, scale scores 20–80) (42). The total STAI-T score varies from 20 to 80. A low score indicates a low level of anxiety, and a high score indicates a high level of anxiety. *QoL* was assessed using WHOQOL-BREF, a 26-item questionnaire rated on a five-point Likert scale for general QoL and 4 subdomains [psychological, physical, social and environmental QoL (43)]. *Anxiety sensitivity* was assessed using the Anxiety Sensitivity Index [ASI, 16 items, scale scores 0–64; three-factorial latent structure (44)]. For questionnaires with test manuals including instructions about how to handle missing values, those instructions were followed when calculating sum scores for respective (sub-)scales (42, 43, 45). For other questionnaires (BDI, ASI), sum scores were calculated if 90% of all items were completed. In these cases, missing values were replaced by the mean value of completed items.

### Calculations

#### Analyses of dropouts

Patients that did vs. did not (*n* = 24) complete post-treatment assessments were compared with respect to their pre-treatment psychopathological symptoms, age and pre-treatment general QoL using *t*-tests and U-test and for demographic variables using Pearson’s Chi-square tests, respectively. We had to exclude patients who had missing sum scores in one of the main outcome scales STAI-T or BDI, or WHOQOL.

#### Analyses of therapeutic changes across treatment

Changes in general QoL across treatment were assessed using a Wilcoxon test since general QoL data did not quit fulfil criteria for interval scale data and did not display normal distribution. For all other outcome variables (i.e., QoL subscales and STAI-T, BDI, and ASI sum scores), *t*-tests were performed to assess changes across treatment. For the ASI, a three-factorial latent structure encompassing the following lower level factors has been used (44): physical concerns (“ASI physical”), mental incapacitation concerns (“ASI mental”), and social concerns (“ASI social”). We performed a confirmatory factor analysis aiming at replicating this latent structure (see Supplementary material for details). Subsequently, we performed *t*-tests comparing respective ASI subscales between pre- and post-treatment to check for therapeutic changes in anxiety sensitivity subcomponents. As within-group treatment effect size we calculated Cohen’s *d* for all variables (±0.2 = small effect, ±0.5 = medium effect, ±0.8 = large effect), except for general QoL where we calculated r with the formula *r* = *z*/sqrt (*n*_*x*_ + *n*_*y*_) as effect size (±0.1 = small effect, ±0.3 medium effect, ±0.5 = large effect) (46). We also evaluated a possible influence of being admitted to the study for an AD, DD or AD/DD on overall psychopathological (i.e., depressive and anxious) symptoms with repeated-measures ANOVAs. Furthermore, groups with different main diagnosis were compared with respect to their general QoL at pre- and post-treatment as well as respective changes across treatment using Kruskal–Wallis-tests.

#### Analyses of relationships between psychopathological symptom changes and changes in quality of life across treatment

To assess correlations between reductions in depressive (Δ BDI) and anxious symptoms (Δ STAI-T) with changes in general QoL (Δ general QoL), Spearman’s correlation coefficients (ρ) were calculated. Correlations between Δ BDI and Δ STAI-T and changes in different subdomains of QoL were assessed using Pearson’s correlation coefficient (*r*) [±0.1 = small effect, ±0.3 = medium effect, ±0.5 = large effect, (46)]. We furthermore statistically compared different subdomains of QoL with respect to their strength of correlation with reductions in depressive and anxiety symptoms using Fisher’s *r*-to *z*-transformation (47, 48). Specifically, we compared correlation coefficients between symptom changes and each specific subdomain of QoL (Δ psychological QoL × Δ BDI and Δ STAI-T *vs.* Δ physical QoL × Δ BDI and Δ STAI *vs.* Δ social relations QoL × Δ BDI and Δ STAI-T *vs.* Δ environmental QoL × Δ BDI and Δ STAI).

#### Prediction of changes in general quality of life

A binary logistic regression analysis was performed to evaluate several predictors with respect to their potential to predict improvement vs. non-improvement in perceived general QoL across treatment. To do so, patients were grouped into individuals displaying improved (i.e., increase by at least one point on the five-point Likert scale) vs. non-improved general QoL across treatment. As predictor variables we entered the following variables: patients’ (1) *age*, (2) *sex*, (3) *education*, (4) *marital status*, (5) presence of any other *comorbid psychiatric diagnosis*, (6) *depressive symptoms at pre-treatment (BDI)* (7) *general QoL at pre-treatment*, as well as (8) *anxiety sensitivity (sum score) at pre-treatment* (ASI). Additionally, we performed three further binary logistic regression models evaluating each of the three ASI lower-order factors (physical, mental, and social components of anxiety sensitivity) as a predictor variable (besides the other seven predictor variables named above). As a measure of fit for the total model we report Nagelkerke’s *R*^2^, respectively. A power calculation was performed on the basis of the regression analysis with a confidence level of 95%.

Entering eight predictors, we estimated a sufficient discriminatory power of the entire model with a sample size of 107 and a statistical test power of 0.8012. Furthermore, it was assumed that at least 20% of the participants would have missing data, so that a minimum number of 128 participants was determined.

All statistical analyses were performed using SPSS 22 (49) software and for the confirmatory factor analysis (CFA) the statistical programming language R (50) with the lavaan package (51) was used.

## Results

### Sample characteristics

From the total sample of 152 patients, 24 patients did only fill in questionnaires at pre-treatment and thus needed to be excluded from further analyses. Importantly, patients that dropped out because of missing post-treatment assessment, did not significantly differ from the rest of the sample in terms of their pre-treatment psychopathological (i.e., depressive or anxious) symptoms [all *t*(150) ≤ -0.51, *p* ≥ 0.614] or their pre-treatment general quality of life (QoL) (*U* = 1223.00, *z* = -1.55, *p* = 0.122). Furthermore, they did not significantly differ with respect to age [*t*(150) = -0.53, *p* = 0.60], sex, marital status, education, main diagnosis for which patients were admitted to the study or being diagnosed with another comorbid disorder (all χ^2^ ≤ 5.62, *p* ≥ 0.060). Of the remaining 128 patients, six patients had missing sum scores in one of the main outcome scales STAI, BDI or WHOQOL and were thus removed from further analyses. Importantly, all main results reported below remain unchanged when including these six participants for those analyses where participants have complete data.

Sociodemographic data of the final study sample of 122 patients are shown in Table 1. The mean age of patients was 36.7 years (SD 12.8) and 67.2% were female. The participants had significant impairments in psychosocial functioning. The educational level was rather low in this sample: 55.7% of participants did not have a secondary school diploma, the unemployment rate was 42.6%, and no partnership was held by 66.4% of all participants.

**TABLE 1:** Sociodemographic data of the study sample.

*N*	122
Sex *n* (% female)	82 (67.2%)
Age *M* (SD)	36.7 (12.8)
Partnership *n* (%)	41 (33.6%)
No partnership *n* (%)	81 (66.4%)
Single	61 (50%)
Divorced	20 (16.4%)
Highest degree *n* (%)	
Pre-secondary school	68 (55.7%)
Secondary school	35 (28.7%)
University	19 (15.6%)
Employed or in training *n* (%)	38 (31.1%)
Retired *n* (%)	13 (10.7%)
Psychopharmacotherapy *n* (%)	118 (96.7%)
Psychiatric treatment *n* (%)	122 (100%)
Psychotherapeutic treatment *n* (%)	122 (100%)

The sample was composed of 33.6% (*n* = 41) of all patients being admitted to the study for an anxiety disorder (AD), 41% (*n* = 50) for a depressive disorder (DD), and 25.4% (*n* = 31) for comorbid anxiety and depression (AD/DD). The frequency of diagnoses of the study sample are shown in Table 2. The sample had predominantly a longer course of illness (participants with a DD had at least two depressive episodes), and more than half of the total participants had at least one comorbid axis 1 diagnosis in addition to the main diagnosis. The level of depressive and anxiety symptoms at the begin of therapy of the three groups (AD, DD, AD/DD) are displayed in Table 3. The DD and AD/DD group showed moderate to severe depressive symptoms and all groups (AD, DD and AD/DD) had high anxiety levels.

**TABLE 2:** Frequency of diagnoses according to ICD-10 in the study sample (comprising AD, AD/DD, and DD group).

Axis I	
Recurrent depressive disorder, *n*	81
Anxiety disorder, *n*	72
Panic disorder without agoraphobia, *n*	14
Agoraphobia without panic disorder, *n*	4
Panic disorder with agoraphobia	45
Social phobia, *n*	16
Generalized anxiety disorder, *n*	28
Hypochondria, *n*	6
Obsessive-compulsive disorder, *n*	37
Posttraumatic stress disorder, *n*	17
Substance abuse disorder, *n*	7

**TABLE 3:** Depressive and anxiety symptoms in the study sample, means and standard errors of the overall BDI and overall STAI-T scores are shown before inpatient psychotherapy.

	AD	DD	AD/DD
*n* (%)	41 (33.6%)	50 (41%)	31 (25.4%)
BDI pre-treatment	18.46 (±1.64)	21.2 (±1.54)	24.52 (±1.96)
STAI-T pre-treatment	56.49 (±1.54)	56.38 (±1.71)	57.45 (±1.71)

BDI, beck depression inventory; STAI-T, state-trait anxiety inventory trait subscale, AD, anxiety disorder; DD, depressive disorder; AD + DD, comorbidity. A total BDI score of 0–9 is considered not depressed, 10–18 indicates mild-moderate depression, 19–29 indicates moderate-severe depression, and 30–63 indicates severe depression. The total STAI-T score varies from 20 to 80. A low score indicates a low level of anxiety and a high score indicates a high level of anxiety. STAI scores of 20–37 are classified as “no or low anxiety,” of 38–44 as “moderate anxiety,” and of 45–80 as “high anxiety”.

### Changes in quality of life and psychopathological symptoms across treatment

**FIGURE 1:**
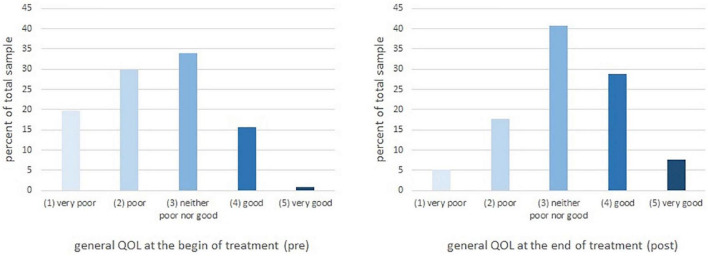
Changes in general quality of life (QoL) across treatment. The proportion of patients from the total sample is displayed on the *y*-axis and the different intervals of general QoL between very poor (1) and very good (5) are displayed on the *x*-axis.

Descriptive data for QoL, anxious and depressive symptoms and anxiety sensitivity at pre-treatment/post-treatment and changes during treatment are displayed in Table 4. For general QoL, a Wilcoxon-test revealed significant improvements with medium effect size from pre-treatment to post-treatment (*z* = -0.587, all *p* < 0.001, all *r* = *0*.38) (Figure 1). A confirmatory factor analysis replicated the three-factorial latent structure of the ASI (anxiety sensitivity index) including physical concerns (“ASI physical”), mental incapacitation concerns (“ASI mental”), and social concerns (“ASI social”) as lower-order factors [see Supplementary material; (44)]. However, even though the three-factorial model properly described the data and almost all items showed significant and large item loadings (i.e., >0.75), two items had somewhat lower loadings (Item 1 and Item 5 of “ASI social” lower-order factor: *“It is important to me not to appear nervous” “It is important to me to stay in control of my emotions“*). Comparing pre- and post-treatment scores in the three ASI lower-order factors, we observed significant improvements across treatment in all ASI subscales (i.e., ASI physical, mental, and social) [all *t*(119) ≥ 2.91, *p* ≤ 0.004, *d* ≥ 0.30].

**TABLE 4:** Changes in quality of life (QoL), psychopathological symptoms, and anxiety sensitivity across treatment descriptive data with relative numbers for general QoL and mean values with standard deviations (SD) for QoL subdomains, anxious/depressive symptoms and anxiety sensitivity at the begin and end of treatment are shown.

	Begin of treatment	End of treatment	*N*	*Z*	*p*	Effect size, *r*
** Changes in general quality of life (QoL)**						
General QoL	“(1) very poor”: 19.8% “(2) poor”: 29.8% “(3) neither poor nor good”: 33.9% “(4) good”: 15.7% “(5) very good”: 0.8%	“(1) very poor”: 5.1% “(2) poor”: 17.8% “(3) neither poor nor good”: 40.7% “(4) good”: 28.8% “(5) very good”: 7.6%	117	–5.87	<0.001*	0.38

	**Begin of treatment** ***M* (*SD*)**	**End of treatment** ***M* (*SD*)**	***df***	***T***	***p***	**Effect size, *d***

** Changes in subdomains of quality of life (QoL)**						
Physical QoL	12.2 (2.82)	14.2 (2.56)	121	–7.31	<0.001*	0.74
Psychological QoL	10.2 (3.02)	12.4 (2.99)	121	–6.89	<0.001*	0.75
Social relations QoL	12.5 (3.52)	12.9 (3.56)	120	–1.17	0.246	0.11
Environmental QoL	13.9 (2.65)	14.6 (2.64)	121	–2.41	0.018*	0.24
** Changes in psychopathological symptoms and anxiety sensitivity**						
Depressive symptoms (BDI)	21.1 (10.92)	12.0 (9.86)	121	8.19	<0.001*	0.87
Anxious symptoms (STAI-T)	56.7 (11.40)	49.0 (10.82)	121	6.38	<0.001*	0.69
Anxiety sensitivity (ASI sum score)	26.29 (14.40)	21.13 (13.15)	119	3.69	<0.001*	0.37

A Wilcoxon-test with its’ effect size *r* for general QoL and t-tests with effect sizes given as Cohens’*d* for QoL subdomains, psychopathological symptoms and anxiety sensitivity are displayed. BDI, beck depression inventory; STAI-T, state-trait anxiety inventory trait subscale; ASI, anxiety sensitivity index. Note that some patients had missing scores for either general QoL, social relations QoL, or ASI leading to slightly diverging degrees of freedom.

*Highly significant.

**FIGURE 2:**
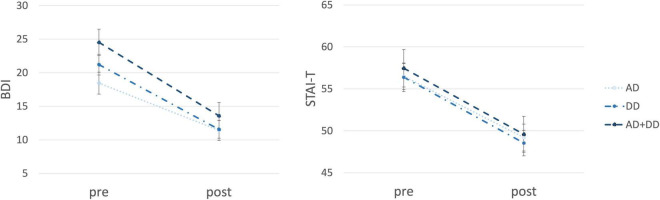
Changes in depressive and anxious symptoms for patients across treatment. On the *y*-axis the symptom severity (BDI/STAI-T) and on the *x*-axis the time (pre/post-treatment) are displayed. The three groups (AD, DD, and AD + DD) are shown by different lines. BDI, beck depression inventory; STAI-T, state-trait anxiety inventory trait subscale; AD, anxiety disorder; DD, depressive disorder; AD + DD, comorbidity. A total BDI score of 0–9 is considered not depressed, 10–18 indicates mild-moderate depression, 19–29 indicates moderate-severe depression, and 30–63 indicates severe depression. The total STAI-T score varies from 20 to 80. A low score indicates a low level of anxiety and a high score indicates a high level of anxiety. STAI scores of 20–37 are classified as “no or low anxiety,” of 38–44 as “moderate anxiety,” and of 45–80 as “high anxiety”.

Evaluating a potential effect of main diagnosis (AD vs. DD vs. comorbid AD/DD) on psychopathological (i.e., anxious and depressive) symptoms, no significant main effects of main diagnosis [all *F*(2,119) < 2.17, *p* > 0.119] and no main diagnosis × time interactions [all *F*(2,119) < 0.97, *p* > 0.381] was found. Thus, results indicate that overall levels of depression and anxiety as well as respective changes across treatment did not significantly differ between patients being admitted for different main diagnosis (Figure 2). Furthermore, patients with different main diagnosis were comparable insofar as patients with AD, DD or AD/DD did not significantly differ with respect to their pre-treatment [χ^2^(2) = 0.45, *p* = 0.800] or post-treatment general QoL [χ^2^(2) = 2.32, *p* = 0.313] or changes in general QoL across treatment [χ^2^(2) = 0.05, *p* = 0.977]. Importantly, significant improvements in general QoL were observed in all three groups with different main diagnosis (all χ^2^ > 3.08, *p* < 0.002).

### Relationships between changes in psychopathological (i.e., anxious and depressive) symptoms and changes in quality of life

For changes in general QoL (Δ QoL), we observed large negative correlations with changes in depressive symptoms (Δ BDI) (ρ = -0.56, *p* < 0.001, *n* = 117 due to missing values for Δ general QoL in five patients; ρ = Spearman’s correlation coefficient, see “Materials and methods” section) as well as changes in anxiety symptoms (Δ STAI) (ρ = -0.49, *p* < 0.001), indicating that improvements in general QoL were strongly related to reductions in psychopathological symptoms. Furthermore, Δ BDI and Δ STAI-T were significantly correlated with changes in all subdomains of QoL, with respective correlation strength, however, varying between different subdomains of QoL (see Table 5).

**TABLE 5:** Correlations between changes in depressive (Δ BDI) and anxious symptoms (Δ STAI-T) and changes in different subdomains of quality of life (Δ QoL) correlation coefficients are displayed.

	Δ Physical QoL	Δ Psychological QoL	Δ Social relations QoL	Δ Environmental QoL
Δ BDI	–0.60*	–0.71*	–0.32*	–0.41*
Δ STAI-T	–0.60*	–0.70*	–0.40*	–0.38*

Pearson correlation coefficients (*r*) are displayed, *p* < 0.001 for all correlations; *N* = 122 for all correlations, except for correlations involving Δ social relations QoL (*n* = 121 due to missing sum score for one patient).

*Highly significant.

### Prediction of changes in general quality of life

We performed a logistic regression analysis predicting whether patients’ perceived general QoL improved during treatment. In total, 52.1% (*n* = 61) reported that their general QoL had improved across treatment (i.e., increase of at least one point on the 5-point Likert scale from pre- to post-treatment). In the remaining 47.9% (*n* = 56) no improvement was observed in perceived general QoL (*n* = 47 of these patients had reported unchanged QoL, *n* = 9 had reported worsened QoL; both groups were treated in one group due to small number of patients with no improvements in QoL). Table 6A displays the results of the main logistic regression analysis testing ASI sum score besides seven other variables as predictors for (non-)improvement in general QoL across treatment. A test of the full model indicated that the predictors as a set reliably distinguished between patients that reported either improved or non-improved general QoL across treatment [χ^2^(9) = 36.06, *p* < 0.001], with Nagelkerke’s *R*^2^ = 0.35 pointing to a large effect. In total, 74.4% of all patients were classified correctly (as not having improved vs. having improved in general QoL) based on the model. A significant contribution to prediction was only found for general QoL at pre-treatment (Wald = 17.89, *p* ≤ 0.001; see Table 6A). More specifically, we found that as general QoL reported at pre-treatment increases, the odds of reporting improved general QoL across treatment decrease (OR = 0.32). In other words, results suggest that improvements in general QoL across treatment are more likely if patients display comparably poor general QoL at the beginning of treatment. Patients’ age, sex, education, marital status, presence of psychiatric comorbidity, depressive symptoms and anxiety sensitivity (sumscore) at pre-treatment did not make any significant prediction for patients’ improvement vs. non-improvement in general QoL across treatment (all Wald ≤ 2.55, *p* ≥ 0.110, Table 6A).

**TABLE 6:** Prediction of improvement vs. non-improvement in general QoL across treatment.

	*B* (SE)	Wald	*P*	Odds ratio	95% CI for odds ratio

(A)					
Intercept	3.46 (1.21)	8.17			
Age	−0.03(0.02)	2.10	0.147	0.97	0.94–1.01
Sex (male vs. female)	−0.29(0.50)	0.35	0.553	0.75	0.28–1.97
**Education**					
Less than secondary school degree vs. university degree	0.87 (0.69)	1.60	0.206	2.39	0.62–9.19
Secondary school degree vs. university degree	0.83 (0.72)	1.33	0.248	2.28	0.56–9.26
Marital status (no relationship vs. in relationship)	0.74 (0.50)	2.20	0.138	2.09	0.79–5.53
Psychiatric comorbidity	−0.23(0.45)	0.25	0.616	0.80	0.33–1.94
BDI pre-treatment	0.02 (0.03)	0.73	0.392	1.02	0.97–1.07
General QoL pre-treatment	−1.14(0.27)	17.89	**< 0.001** *	0.32	0.19–0.54
ASI (sum score) pre-treatment	−0.03(0.02)	2.55	0.110	0.97	0.94–1.01

**(B)**					

Intercept	4.72 (1.41)	11.20			
Age	−0.03(0.02)	3.18	0.074	0.97	0.93–1.00
Sex (male vs. female)	−0.12(0.49)	0.06	0.806	0.89	0.34–2.33
**Education**					
Less than secondary school degree vs. university degree	0.70 (0.66)	1.12	0.289	2.01	0.55–7.33
Secondary school degree vs. university degree	0.46 (0.70)	0.43	0.511	1.59	0.40–6.30
Marital status (no relationship vs. in relationship)	0.80 (0.50)	2.57	0.109	2.24	0.84–5.97
Psychiatric comorbidity	−0.18(0.46)	0.16	0.694	0.83	0.34–2.07
BDI pre-treatment	0.03 (0.02)	1.05	0.305	1.03	0.98–1.07
General QoL pre-treatment	−1.13(0.27)	16.90	**< 0.001** *	0.33	0.19–0.56
ASI (item 1 and 5) pre-treatment	−0.32(0.14)	5.25	**< 0.022** *	0.73	0.55–0.96

(A) Variables (age, sex, education, marital status, psychiatric comorbidity, BDI, general QoL, and ASI sum score) and parameters (incl. odds ratios) of the regression analysis are shown. *R*^2^ (Nagelkerke) = 0.35, Model χ^2^(9) = 36.06, *p < 0.001. The model is based on 117 patients due to missing values for general QoL for pre- or post-treatment in five patients. BDI, beck depression inventory; ASI, anxiety sensitivity index.

(B) Variables (age, sex, education, marital status, psychiatric comorbidity, BDI, general QoL and ASI item 1 and 5) and parameters (incl. odds ratios) of the regression analysis are shown. *R*^2^ (Nagelkerke) = 0.38, Model χ^2^(9) = 39.18, *p < 0.001. The model is based on 117 patients due to missing values for general QoL for pre- or post-treatment in five patients. BDI, beck depression inventory; ASI, anxiety sensitivity index. Item 1 and Item 5 of ASI social lower order factor: “*It is important to me not to appear nervous*” “*It is important to me to stay in control of my emotions*“).

Finally, following up on the results of the confirmatory factor analysis for the ASI (see above), we performed another exploratory regression analysis testing whether the two ASI items that showed lower factor loadings as compared to the other ASI items could predict (non-) improvement in general QoL. To do so, we entered the same predictor variables as above and additionally included a sum score of ASI item 1 and 5 (*“It is important to me not to appear nervous”* and *“It is important to me to stay in control of my emotion“*; both items belong to the lower order factor “ASI social”) as additional predictor variable (see Table 6B). Again, the binary regression analyses yielded that the predictor variables as a set reliably distinguished between patients that reported improved or non-improved general QoL [all χ^2^(9) ≥ 39.18, *p* ≤ 0.001, Nagelkerke’s *R*^2^ ≥ 0.38]. In total, 75.2% of all patients could be classified correctly based on the model. We found a significant contribution to prediction for general QoL at pre-treatment (Wald = 16.90, *p* < 0.001). Additionally, we found a significant contribution of ASI item 1 and 5 (Wald = 5.25, *p* = 0.022). More specifically, we found that as general QoL (OR = 0.33) and scores for ASI items 1 and 5 (OR = 0.73) reported at pre-treatment increase, the odds of reporting improved general QoL across treatment decrease, i.e., improvements in general QoL across treatment are more likely in patients with a comparably low general QoL and a low desire not to appear nervous and to stay in control of one’s emotions at pre-treatment. Again, patients’ age, sex, education, marital status, presence of psychiatric comorbidity and depressive symptoms at pre-treatment did not make any significant prediction for patients’ (non-)improvement in general QoL (all Wald ≤ 3.18, *p* ≥ 0.074 see Table 6B).

## Discussion

Anxiety and depressive disorders (AD and DD) are associated with dysfunctional thoughts that prevent patients from achieving their goals or living a fulfilling life. Cognitive behavior therapy (CBT) helps to identify and challenge unhelpful thoughts, which on the one hand should directly influence disorder-specific thoughts but on the other hand might also lead beyond or more directly to positive changes in quality of life (QoL).

This study investigated associations between improvements of clinical symptoms and changes in QoL of inpatient psychotherapy patients. In a second step predictors for improvements in QoL were identified.

The subjective level of QoL is an important indicator of response to psychotherapy because it can detect positive changes in cognitively mediated perceptions of life satisfaction, despite persisting clinical symptoms or an unchanged social environment. In line with previous research, results demonstrate moderate effects of the inpatient CBT on general QoL for patients with the main diagnosis AD and/or DD. Surprisingly, only half of all patients reported improvements in their general QoL, whereas the other half failed to do so. No satisfactory explanation was found for this trend, different types of the main diagnoses and comorbid psychiatric diagnoses did not influence these outcomes.

In contrast to these findings, comorbidity of AD and DD is described to be associated with detriments in psychosocial functioning and QoL in literature (52) maybe due to different levels of severity of clinical symptoms or other circumstances not evaluated in the present study.

Positive changes in clinical symptoms were significantly correlated with changes in general QoL, a finding which has been well established in existing studies (53, 54). Looking at correlations between changes in clinical symptoms and changes in specific domains of QoL, strong correlations were found only for the psychological and physical domains of QoL. For the environmental and social domains only small correlations were detected. Similarly, medium to strong effect sizes of improvements following CBT were shown for QoL in the psychological and physical domain but only small effects on the environmental domain. When looking for the social domain, no significant changes were reported. These findings are similar to those described in a meta-analysis, where beneficial effects of CBT on QoL for patients with AD were found, but particularly in the physical and psychological domains (27).

A prospective study (18) compared the effectiveness of 3 different therapeutic methods (supportive-expressive therapy, antidepressant medication and placebo for 16 weeks) in improving QoL in patient groups with AD/DD. Results showed that distinct treatments for symptoms of depression and anxiety worked equally well regardless of the specific outcome being measured. Additionally, evaluation of their study showed that patients experienced not only a significant reduction in depressive and anxiety symptoms, but also a significant improvement in many other aspects of life, including social relationships. This is different to the results of the present study showing no significant improvement in the social domain, maybe due to a shorter treatment period (8 weeks) compared to a 16-weeks program (18).

Only about half of all patients reported improvements in their general QoL in this study, the other half failed to do so in the present study. A German study evaluated changes between partly, fully and non-remitted depressive patients and found significant group differences only for the domains overall QoL, physical and psychological health. Similar to the results of the present study, no significant differences were reported for social and environment domain—indicating that the severity of clinical symptoms only partly determines subjective QoL. An Italian study hypothesized that the presence of subthreshold panic-agoraphobic symptomatology in otherwise healthy individuals significantly impaired QoL despite the absence of a full-blown PD diagnosis (55).

The presence of an intimate partnership was the only factor associated with high levels of QoL in the psychological and in the social relationship domains in a German study (56). Two thirds of the patients interviewed for the present study were not living in a partnership, a factor not easily to be changed. This might be one explanation for patients not improving much in social domains of QoL during the program.

Clinical symptoms improve faster than psychosocial functioning, which takes longer to improve and moderate psychosocial impairments may remain. Similar to our results, a meta-analysis found that psychotherapy for DD leads to improvements in social functioning but less than for depressive symptoms (57). The authors argued that changes in social functioning cannot be fully explained by changes of depressive symptoms and that other factors do also play important roles. Clinical improvement, therefore, seems to have only little immediate impact on social functioning, at least for patients with AD and/or DD. Distortions in social life persist even when symptoms have subsided, and 8 weeks may be too short a time for environmental and social changes.

When looking at possible predictors for improvements in QoL, a regression analysis was performed and results showed that improvements in general QoL across treatment are more likely if patients reported comparably poor general QoL at baseline, so there might be enough room and possibility for improvements during the 8-weeks treatment program.

A second interesting finding was that improvements in general QoL are more likely for patients who reported less anxiety to appear nervous in front of others or to lose control of their emotions (according to items 1 and 5 of the ASI questionnaire). These results were the same for all three diagnostic groups. Our results suggest that for patients with signs of social anxiety these negative cognitions seem to be of high importance for treatment-associated changes in QoL. To our knowledge this is the first study examining the influence of anxiety sensitivity on treatment response in terms of QoL in a diagnostic sample of patients with AD and/or DD. An explanation might be subthreshold symptoms of social anxiety disorders (compared to a control group) which are described as persisting and impairing conditions, resulting in considerable subjective suffering and negative impact on social relationships with no significant differences compared to the pure diagnostic group (58).

Anxiety sensitivity social concerns is a dimension of anxiety sensitivity and reflect the tendency to fear potential negative evaluations resulting from others noticing symptoms of anxiety, such as sweating or blushing. One explanation for the present results might be that high AS at baseline, especially for items 1 and 5, discourages patients to talk about their symptoms in front of others resulting in less social contacts and less improvements in social QoL. Also, the cognitive concerns of anxiety sensitivity with strongly held beliefs that anxiety symptoms are dangerous and must be controlled may result in lower treatment engagement due to these fears.

One could conclude that patients who are more anxious about showing their nervousness in front of others may need special encouraging techniques to improve their social skills and self-esteem in order be more comfortable in social situations, even when possibly evaluated by others. A more individualized psychotherapy approach focusing on improvements in self-acceptance and emotional competence should an important future treatment approach for this group of patients. Therefore, we suggest social skills training (59) for these patients so that they learn to open up in front of others to benefit from (group) psychotherapy in an initially helpful way. Krumholz et al. (60) found that patients with anxiety and depression particularly showed difficulties with interpersonal skills, which may be due to deficits in understanding or performing social skills or a combination of both.

Social skills training is particularly indicated for individuals who have not learned appropriate interpersonal skills or have difficulty recognizing and understanding subtle cues in social interactions. Social skills training is a type of behavior therapy and includes interventions and teaching methods that help individuals improve and understand their social behavior and aims to teach people verbal and non-verbal behaviors that occur in typical social interactions (56).

## Conclusion

AD and DD are associated with dysfunctional thoughts that prevent patients from achieving their goals or living a fulfilling life, so they are associated with significant impairment in QoL across multiple domains. CBT helps identify and address unhelpful thoughts, which can lead to improvement in clinical symptoms, but might also directly lead to positive changes in QoL. Therefore, subjective levels of QoL are an important indicator of response to psychotherapy, as positive changes in cognitively mediated perceptions of life satisfaction can be detected, but most of the research on psychotherapy outcomes has focused on symptom reduction as the main outcome variable. A positive effect of psychotherapy on symptom reduction and QoL improvement has been demonstrated in some studies, but evidence on the relationship between these two treatment goals is sparse and inconclusive, and predictors of positive treatment outcome in terms of QoL have not been adequately explored.

In the current study we firstly, investigated the associations between improvements of clinical symptoms and QoL in individuals with AD and/or DD after an inpatient CBT program and secondly, looked for possible predictors for QoL improvements after CBT. Firstly, the inpatient CBT program showed moderate to strong effects on general QoL and psychopathological symptoms, and changes in symptom burden correlated most strongly with psychological and physical QoL. Our results suggest that clinical symptoms and general QoL improve more rapidly than environmental or even social subdomains of QoL. This is reflected in the psychosocial functioning of patients, which often does not improve for some time after symptom remission (61), and moderate psychosocial impairments may persist even after short-term treatments.

Secondly, a positive treatment outcome in terms of general QoL improvements were more likely in patients with low general QoL at pre-treatment and in patients with less anxieties to appear nervous or to lose control of emotions. Individuals with poor QoL who have fewer inhibitions about openly expressing their anxious feelings in (individual and group) psychotherapy benefit particularly from CBT in terms of improving their QoL. In contrast, our research findings suggest that patients who are too anxious to openly express their nervousness should receive additional, more targeted treatment to build sufficient self-confidence to better engage in the treatment program. Therefore, we suggest social skills training (59) for the patients to help them learn to open up in front of others in order to benefit from (group) psychotherapy in an initially helpful way.

There are still some questions about the impact of change on QoL for people with anxiety and depression. To enhance our understanding of improvements in QoL after psychotherapy, comparative studies with longer follow-up periods are needed to examine the long-term effects of psychotherapy on QoL. A better understanding of changes in specific QoL subdomains following evidence-based psychotherapy would lead to further improvements in the effectiveness of interventions for AD and DD.

## Limitations

The results must be interpreted with care due to the lack of a control group for comparison of treatment outcome. Results for predictors for improvements in QoL should be interpreted with caution until their replication due to the sufficient but rather small sample size of our study and the heterogeneity of the patients.

Patients were assigned to the three groups (AD, DD, AD/DD) according to their ICD-10 diagnosis, but the degree of severity of depression or the type of anxiety disorder was not considered. The composition of the groups could possibly lead to distortions of the results.

The inpatient psychotherapy program was intensive but short-term (8 weeks). The endpoint was fixed at the beginning of the therapy, which certainly influenced the psychotherapeutic process. The time limit may create an expectation pressure and influence the attitude of the patient and the therapist (62). Another important limitation is the lack of follow-up assessments. Follow-up studies are necessary to learn more about possible long-term effects of psychotherapy on QoL with its’ subdomains. Therefore, a follow-up period of 3, 6, or 12 months might be interesting. In our study some patient’s social problems endure even when clinical symptoms have stopped and several QoL domains improved. This might have resulted from the shortness of the psychotherapy program and the assessment period. To look if social QoL domain will improve in the future and if changes in other QoL domains last or return to previous levels, follow-up studies are necessary to find out more about the long-term effects.

The results of the present study on inpatient treatment with a focus on CBT can certainly be transferred to the results of outpatient treatment, but with a limited extent. One study in a smaller sample of outpatients with ADs found similar results for QoL improvements (31). But patients in inpatient psychotherapy are on average more severely ill or have a lower level of psychosocial functioning than patients in outpatient treatment, who often still have a job or other family responsibilities, which is more compatible with outpatient treatment. Our sample had a high rate of comorbidities, rather long courses of illness, a rather high level of psychopathological symptoms, and significant impairments in psychosocial functioning levels (especially work and partnership).

Inpatient psychotherapy also differs from outpatient therapy in several aspects as the inpatient setting creates a protected environment for several weeks with distance to the private and professional environment under which some problems may appear from different lights. The lack of immediate demands from real life can also contribute to improvement but allows an in-depth concentration on oneself. The treatment offered in inpatient therapy is multimodal and intensive. Group and individual therapy offer contact with various therapists and fellow patients. Due to the intensive preoccupation with the problems of fellow patients, inpatient stays can also result in patients with poor dissociation strategies sometimes feeling heavily burdened.

## Data availability statement

The datasets presented in this article are not readily available due to privacy and ethical concerns. Access to the data can be provided after anonymization and testing for compliance with the privacy policy. Requests to access the datasets should be directed to gruber.maria@pm.me.

## Ethics statement

The study was approved by the ethics committee of the Medical University of Vienna (EK Nr: 1933/2016). The authors assert that all procedures contributing to this work comply with the ethical standards of the Medical University of Vienna committee in accordance with the Helsinki Declaration of 1975, as revised in 2008. Written informed consent for participation was not required for this study in accordance with the national legislation and the institutional requirements.

## Author contributions

MF designed the study. MF and MG conducted the study, partially analyzed the data, and wrote this manuscript. MW performed the main part of data analysis, wrote the manuscript partly, and provided feedback on each phase of the research, including a full review and feedback on the final manuscript. RWA performed the CFA and provided statistical support. FP-M helped with writing the manuscript, particularly with reference management. ZL and DK assisted with data acquisition and provided feedback on the manuscript. All authors contributed to the article and approved the submitted version.
