# Supplementary material for: Determinants of quality of life improvements in anxiety and depressive disorders—A longitudinal study of inpatient psychotherapy
Source: Front Psychiatry. 2022 Dec 15;13:937194. doi: 10.3389/fpsyt.2022.937194 (PMC9798124; doi:10.3389/fpsyt.2022.937194)
Supplement: Supplementary file 1 [file Data_Sheet_1.pdf]

Supplementary material to

Determinants of quality-of-life improvements in anxiety and depressive disorders—A longitudinal study of inpatient psychotherapy

Marion Freidl, Melanie Wegerer, Zsuzsa Litvan, Daniel König, Rainer W. Alexandrowicz, Filipe Portela-Millinger and Maria Gruber

frontiers in Psychiatry: DOI 10.3389/fpsyt.2022.937194

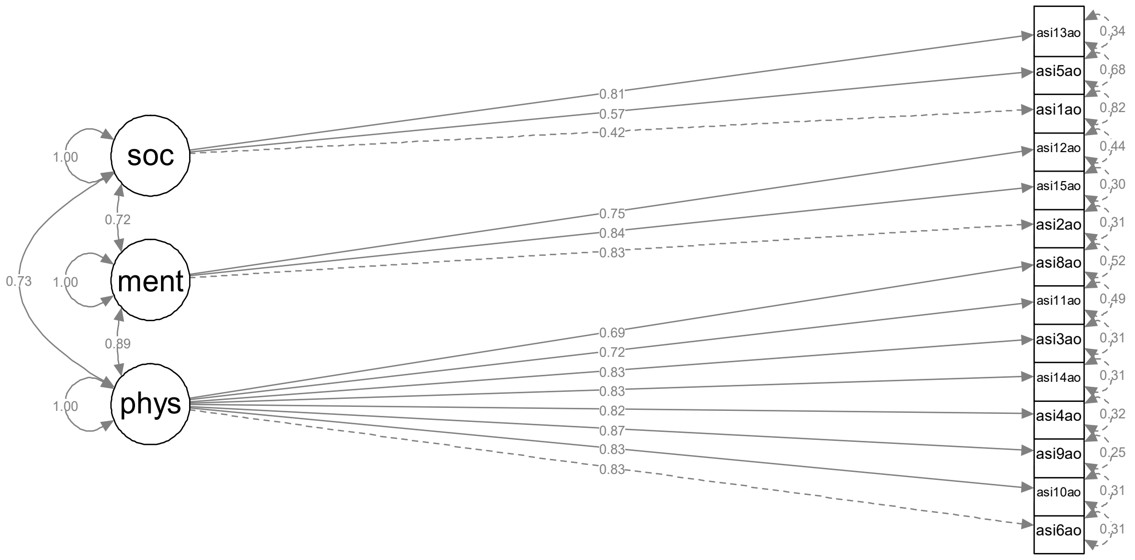

Figure S 1: Diagram of the Confirmatory Factor Analysis at admittance.

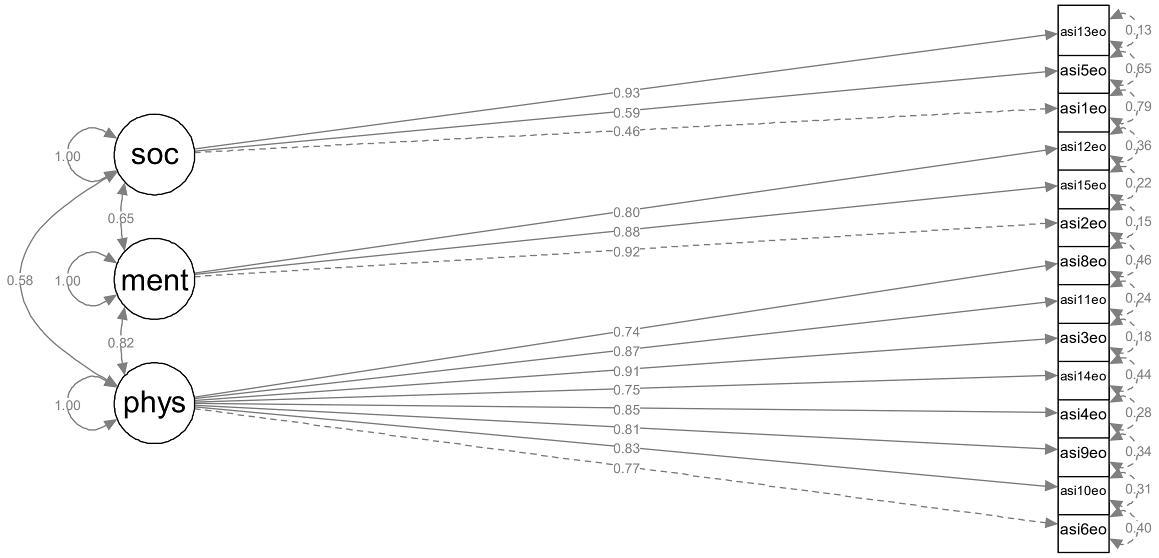

Figure S 2: Diagram of the Confirmatory Factor Analysis at dismissal.

Table S1 Latent Variables of items at admittance

	Estimate	Std.Err	z-value	P(>|z|)	Std.lv	Std.all
**Physical concerns**						
asi6ao	1.000				0.833	0.**833**
asi10ao	0.996	0.049	20.208	0.000	0.829	**0.829**
asi9ao	1.040	0.049	21.395	0.000	0.866	**0.866**
asi4ao	0.989	0.046	21.637	0.000	0.823	**0.823**
asi14ao	0.996	0.047	21.133	0.000	0.829	**0.829**
asi3ao	0.999	0.045	22.179	0.000	0.832	**0.832**
asi11ao	0.860	0.081	10.676	0.000	0.716	**0.716**
asi8ao	0.829	0.065	12.821	0.000	0.690	**0.690**
**Mental concerns**						
asi2ao	1.000				0.828	**0.828**
asi15ao	1.009	0.071	14.116	0.000	0.836	**0.836**
asi12ao	0.903	0.071	12.682	0.000	0.748	**0.748**
**Social concerns**						
**asi1ao**	**1.000**				**0.425**	**0.425**
**asi5ao**	**1.337**	**0.350**	**3.822**	**0.000**	**0.568**	**0.568**
asi13ao	1.904	0.438	4.351	0.000	0.809	**0.809**

Table S2 Latent Variables of items at dismissal

	Estimate	Std.Err	z-value	P(>|z|)	Std.lv	Std.all
**Physical concerns**						
asi6eo	1.000				0.772	**0.772**
asi10eo	1.076	0.061	17.665	0.000	0.831	**0.831**
asi9eo	1.051	0.069	15.212	0.000	0.811	**0.811**
asi4eo	1.099	0.063	17.359	0.000	0.849	**0.849**
asi14eo	0.966	0.062	15.562	0.000	0.746	**0.746**
asi3eo	1.173	0.059	19.929	0.000	0.906	**0.906**
asi11eo	1.129	0.063	17.854	0.000	0.872	**0.872**
asi8eo	0.954	0.063	15.074	0.000	0.736	**0.736**
**Mental concerns**						
asi2eo	1.000				0.921	**0.921**
asi15eo	0.956	0.072	13.326	0.000	0.880	**0.880**
asi12eo	0.871	0.053	16.312	0.000	0.802	**0.802**
**Social concerns**						
**asi1eo**	**1.000**				**0.461**	**0.461**
**asi5eo**	**1.276**	**0.260**	**4.916**	**0.000**	**0.589**	**0.589**
asi13eo	2.023	0.448	4.517	0.000	0.933	**0.933**

Table S3 Fit Measures (Both Models Admittance and Dismissal)

	**Admittance**	**Dismissal**
χ2	119.022	161.320
df	74.000	74.000
P	0.001	0.000
CFI	0.993	0.987
TLI	0.991	0.984
RMSEA	0.070	0.098
RMSEA.ci.lower	0.046	0.078
RMSEA.ci.upper	0.093	0.119
RMSEA.p	0.081	0.000
RMR	0.073	0.085
SRMR	0.077	0.090
GFI	0.985	0.982
